# Phenotypic and Genotype Patterns of Antimicrobial Resistance in Non-Human Primates: An Overlooked “One Health” Concern

**DOI:** 10.3390/antibiotics14100985

**Published:** 2025-09-30

**Authors:** Juan Wen, Samuel Kumi Okyere, Yujie Shi, Yu Qu, Chaoxi Chen

**Affiliations:** 1 Chengdu Zoo & Chengdu Research Institute of Wildlife, Chengdu 610081, China; juanwen881010@163.com (J.W.); walnut0215@163.com (Y.S.); quyu1120_hao@163.com (Y.Q.); 2 Key Laboratory of Animal Disease and Human Health of Sichuan Province, Sichuan Agricultural University, Chengdu 611130, China; 2019603005@stu.sicau.edu.cn; 3 College of Animal and Veterinary Sciences, Southwest Minzu University, Chengdu 610041, China

**Keywords:** non-human primates, antimicrobial resistance, bacteria, zoonosis, one health approach

## Abstract

Non-human primates (NHPs) are close relatives of humans and can serve as hosts for many zoonotic pathogens. They play crucial role in spreading antimicrobial resistant bacteria (AMR) to humans across various ecological niches. The spread of antimicrobial resistance in NHPs may complicate wildlife conservation efforts, as it may threaten domestic livestock, endangered species as well as human’s health. This review analyses the existing literature on the prevalence of AMR in NHP species, including *Rhinopithecus roxellana*, *Macaca fascicularis*, and *Sapajus nigritus*, to create awareness in all stake holders involve in the fight against AMR on the serious potential threats that these primates pose. Methods: We performed a comprehensive literature search using the PubMed (National Library of Medicine-NLM), Scopus (Elsevier), Web of Science Core Collection (Clarivate Analytics), Springer Link (Springer), and Science Direct (Elsevier) databases until January, 2025. The search strategy combined terms from the areas of non-human primates, antibiotic resistance, antimicrobial resistance, and antibacterial resistance genes (ARGs). Studies that isolated bacteria from NHPs and assessed phenotypic resistance to specific antibiotics as well as studies that identified ARGs in bacteria isolated from NHPs were included. Data were synthesised thematically across all included studies. Results: A total of 37 studies were included (explained as Cercopithecidae (*n* = 23), Callithrix (*n* = 6), Cebidae (*n* = 4), Hominidae (*n* = 3), and Atelidae (*n* = 1)). The results showed that the most common ARB across the various NHPs and geographical settings was *Staphylococcus* spp. (45.95%) and *Escherichia* spp. (29.73%). The tested antibiotics that showed high levels of resistance in NHPs included Tetracycline (40.54%), Ciprofloxacin (32.43%), and Erythromycin (24.34%), whereas ermC, tetA, tetM, aadA, aph (3″)-II, and qnrS1 were the most widely distributed antibiotic resistance genes in the studies. Conclusion: NHPs are potential natural reservoirs of AMR, therefore global policy makers should consider making NHPs an indicator species for monitoring the spread of ARB.

## 1. Introduction

Antibiotics have been used extensively in contemporary medicine, agriculture, and livestock farming methods for a number of decades. They are employed as growth promoters to improve the growth and feed conversion efficiency of food animals or to prevent and treat bacterial infections in both humans and animals [1,2]. The development and spread of antibiotic-resistant bacteria (ARB) and antibiotic resistance genes (ARGs) are facilitated by the accumulation of antibiotics, which are emerging environmental pollutants, in animal bodies and environmental media [3]. Antimicrobial resistance (AMR) has quickly grown to be a major global public health concern and threat, as it has the potential of diminishing the therapeutic potential of antibiotics against a variety of pathogenic microorganisms in humans and animals [4].

The development of AMR is an evolutionary outcome of microbial competition, but the extreme and inappropriate use of antibiotics in clinical and non-clinical settings (misuse and overuse) has exacerbated this process. AMR can spread across all ecosystems, such as farms, hospital discharges (human and veterinary), and landfills [5]. Selective stress of antibiotics is beneficial to ARB, which are transmitted through human and animal (wild or domestic) activities, including human-induced processes such as trade of animals and their products, as well as natural periodic migratory activities of wildlife (e.g., birds) [6,7]. Furthermore, phage-mediated horizontal gene transfer or mobile genetic elements (plasmids, transposons, integrons, and integron-associated elements) can transfer ARGs to host-associated and environmental microbial communities, making disease treatment and new pollutant management more difficult [8]. It is well acknowledged that plasmids are mobile vectors that carry ARGs in clinical pathogens [9]. Plasmids are capable of moving between bacteria and are recognised as being important vehicles that transfer antibiotic resistance genes (ARGs) between bacterial species [9,10]. Transposons and other mobile genetic elements facilitate horizontal gene transfer in the gut microbiota, allowing some pathogenic bacteria to acquire antibiotic resistance genes (ARGs) [11]. Therefore, efficient ways to track and manage the spread of ARBs and ARGs should be examined within the framework of the “One Health” Joint Plan of Action (2022–2026).

Humans, animals, and wildlife, including non-human primates (NHPs), act as reservoirs and vectors for ARBs and ARGs [12,13]. The likelihood of NHPs coming into contact with humans through shared environments or direct interaction has increased in recent years due to factors such as habitat fragmentation, poaching, the “bush meat” trade, and (ecological) tourism, even though the majority of NHPs prefer to live in areas that are not densely populated. This is especially true when the animals are transferred to zoos for ex situ conservation or admitted to wildlife rescue centres for clinical treatment [14]. NHPs also share high similarities with humans in terms of neuroanatomy, organ structure, physiology, and social behaviour. This close phylogenetic relationship between human and NHPs may predispose them to colonisation or infection by species that are pathogenic to humans or the same species [15]. Therefore, NHPs are unique and serve as excellent sentinels for monitoring zoonotic transmission and epidemics. Numerous studies have reported the identification of ARMs and ARGs from NHPs [16]. However, in captivity, one of the major challenges for many veterinarians who work with NHPs is the lack of documentation and information about the prevalence and effects of ARB from most science centres [17,18]. Since NHPs serve as potential threats to public health, there is a need to provide concrete information about their threats to create awareness and encourage all stake holders involved in the fight against AMR to pay more attention to these animal species. Therefore, in order to provide veterinarians, carers, and researchers working on NHPs with helpful resources in the literature, this review attempts to identify and describe scientific reports pertaining to antimicrobial resistance phenotypes and genotypes in NHPs. Understanding the epidemiology of AMR in NHPs is essential for developing and refining intervention strategies and taking proactive measures to contribute to the protection of NHPs and public health. Finally, this review provides an outlook on the current status of research into phenotypic and genotypic patterns of antimicrobial resistance in NHPs, including the use of technologies such as whole-metagenome sequencing to measure antimicrobial resistance, as well as the establishment of NHP bacterial resistance monitoring systems in developing countries.

## 2. Results

### 2.1. Descriptive Statistics of Included Studies

**Figure 1:**
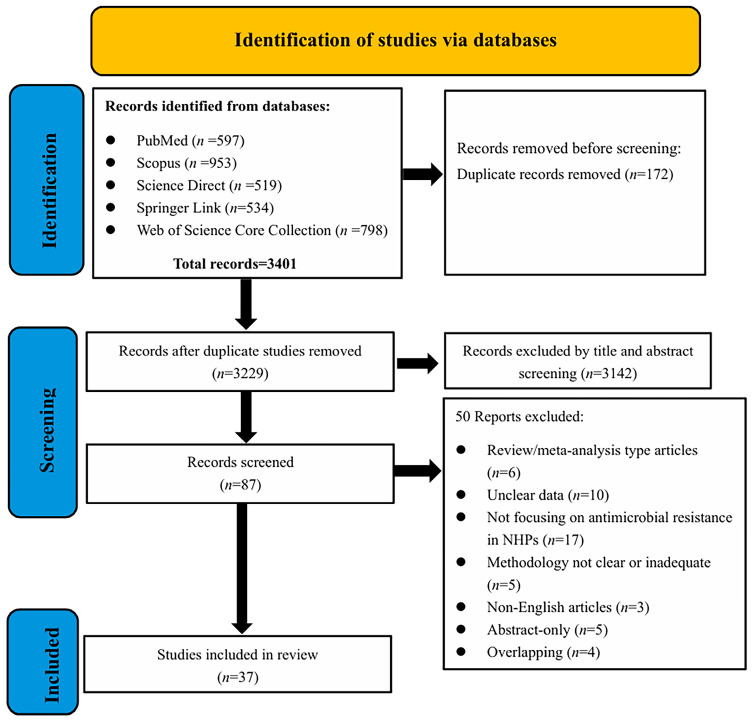
PRISMA flow diagram of study selection procedure.

The PRISMA method identified a total of 3401 records from the following sources: PubMed *(n* = 597), Web of Science Core Collection (*n* = 798), Scopus (*n* = 953), Science Direct (*n*= 519), and Springer Link (*n* = 534). After excluding duplicate records (*n* = 172), the remaining records totaled 3229. By reviewing the titles and abstracts for relevance, we excluded irrelevant studies (*n* = 3142) because they did not cover the phenotypic and genotypic patterns of antimicrobial resistance in non-human primates (NHPs). The remaining 87 articles were assessed for eligibility based on the existing inclusion and exclusion criteria. Among these, 50 articles were excluded for the following reasons: review articles, conference abstracts, lack of NHP samples, and non-open access full texts. Ultimately, only 37 articles were included in this review (Figure 1).

### 2.2. Antibiotic Resistance Studies in NHPs

NHPs refers to primates other than humans, belonging to the class Mammalia and order Primates [19]. They can be found in various habitats, including biodiverse tropical rainforests, swamp forests, savannas, urban areas, and mountainous regions with desert or arid conditions [20]. Research on antimicrobial resistance in NHPs has primarily focused on the *Cebinae*, *Callithrix*, *Hominidae*, *Cercopithecidae*, and *Atelidae* families.

#### 2.2.1. Basic Characteristics of Selected Studies

**Figure 2:**
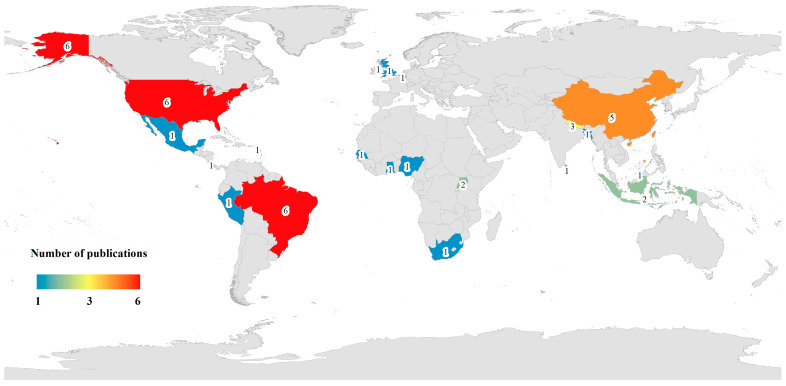
The national/regional distribution of global NHPs antimicrobial resistance research publications from 2015 to 2025.

The 37 qualified and analysed journal articles included in the study were ranked in descending order as follows: *Cercopithecidae* 62.16% (*n* = 23), *Callithrix* 16.22% (*n* = 6), *Cebidae* 10.81% (*n* = 4), *Hominidae* 8.11% (*n* = 3), and Atelidae 2.7% (*n* = 1). The aforementioned journal articles encompassed antibiotic resistance data for NHPs from 20 countries across 6 continents, specifically, Brazil, the USA, China, Nepal, England, Indonesia, Senegal, Uganda, Costa Rica, Belgium, Peru, Brunei Darussalam, Sri Lanka, Bangladesh, South Africa, Nigeria, Saint Kitts and Nevis, Ghana, Gambia, and Mexico (Figure 2). Approximately 32.43% (*n*/*N* = 12/37) of the articles were conducted in Brazil and the USA, whereas 13.51% (*n*/*N* = 5/37) were conducted in China. All the journal articles included in this review provide crucial data on obtaining ARB and ARGs from NHPs (Table 1, Table 2, Table 3, Table 4 and Table 5).

#### 2.2.2. Prevalence of Antimicrobial Bacteria in NHPs

**Figure 3:**
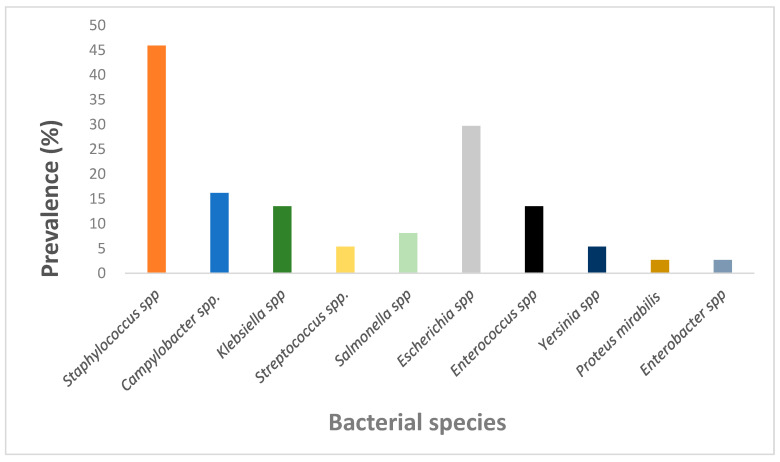
Prevalence of bacterial genera in antimicrobial resistance studies in NHPs.

This systematic review primarily covers 10 bacterial genera (*Staphylococcus* spp., *Campylobacter* spp., *Klebsiella* spp., *Streptococcus* spp., *Salmonella* spp., *Escherichia* spp., *Enterococcus* spp., *Yersinia* spp., *Proteus mirabilis* and *Enterobacter* spp. Among them *Staphylococcus* spp., *Escherichia* spp., *Campylobacter* spp. and *Enterococcus*/*Klebsiella* spp. showed the most prevalence, at 45.95%, 29.73%, 16.22% and 13.51%, respectively (Figure 3). *Staphylococcus* spp. were prevalent among three out of the five NHP species, whereas *Escherichia coli* was identified in four out of the five NHP species, indicating their higher occurrence in NHPs.

#### 2.2.3. Prevalence of Antibiotic Resistance in NHPs

**Figure 4:**
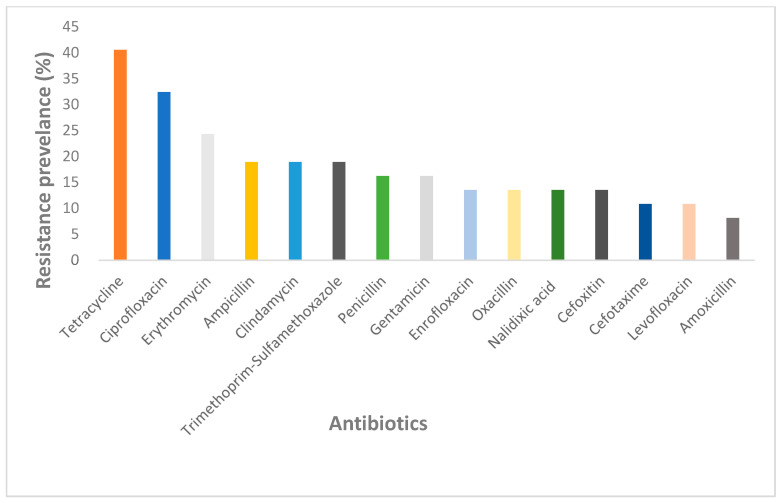
Prevalence of antibiotic resistance phenotypes of top 15 antibiotics in bacteria from NHPs.

A total of 89.19% (n/N = 33/37) of the articles conducted antibiotic susceptibility tests on the isolated and identified bacteria (Table 1, Table 2, Table 3, Table 4 and Table 5). The NHPs *Cebidae* (n = 4), *Callithrix* (n = 6), *Hominidae* (n = 3), and *Cercopithecidae* (n = 20) exhibited resistance to the tested antibiotics. Among the 53 test antibiotics, the antibiotics that showed high levels of resistance in NHPs included Tetracycline (40.54%), Ciprofloxacin (32.43%), Erythromycin (24.34%), and Ampicillin, Clindamycin, and Trimethoprim-Sulfamethoxazole which all had a resistance of 18.92%. (Figure 4). In addition, bacteria resistance of Ciprofloxacin antibiotics was observed in four out of four of the NHP species, whereas resistance of Erythromycin and Tetracycline were only observed in three out of four and two out of four NHP species, respectively.

#### 2.2.4. Prevalence of Antibiotic Resistance Genes in NHPs

**Figure 5:**
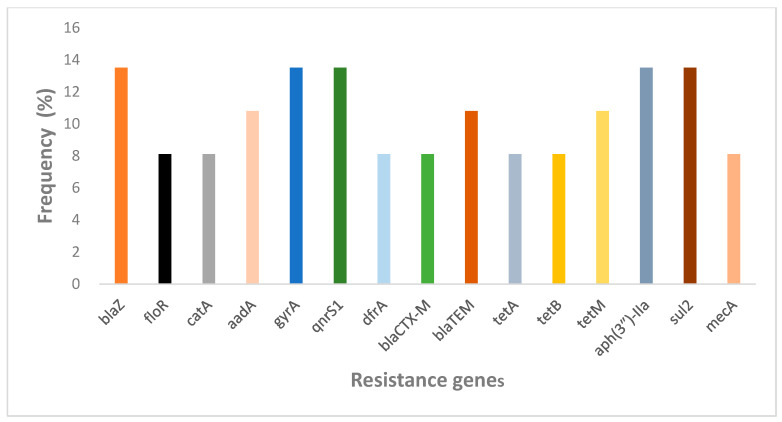
Prevalence of antibiotic resistance genes identified in NHPs.

A total of 56.76% (n/N = 21/37) of the articles reported on resistance genes, primarily identified through PCR, WGS, and DNA microarray analysis. The presence of these genotypes was associated with antibiotic resistance in different categories of antibiotic phenotypes. However, due to the varying methods used to identify resistance genes, obtaining a combined prevalence rate for antibiotic resistance genes posed significant challenges. Therefore, the overall frequency of the top 15 resistance genes was summarised as a bar graph in Figure 5. This includes macrolides (*ermC, mphC, msrA, msrC,* and *mreA*), tetracyclines (*tetA, tetB, tetM, tetO, tetL, tetS, tetK,* and *tet38*), β-lactams (*bla_SHV_, bla_TEM_, blaZ, blaEC, bla_OXA_, bla_CTX-M_, bla_CTM-X_, bla_CMH-3_, bla_ACT-6_, and bla_CMG1_*), methicillin (*mecA*), chloramphenicol (*floR, catA, catB*, and *cmlA1*), fluoroquinolones (*qnrA, qnrB, oqxA, oqxB, qnrS1, qnrD, gyrA, and acrB*), sulfonamides (*sul1, sul2, sul3, dfrA*, and *dfrG*), aminoglycosides (*aacA-aphD*, *aac(3″)-IId, aadA, aph(3″)-IIa,* and *aph(6″)-Id*), and fosfomycin (*fosA*). The results indicated that the resistant genes *blaZ* (β-lactams), *qnrS1* and *gyrA* (fluoroquinolones), *sul2* (sulfonamides), and *aac(3″)-IId* (aminoglycosides) were high and tet family genes (tetracyclines) were widely distributed, which confirms the antibiotic resistance phenotypic results. The sul2 antibiotic resistant gene was identified in four out of five NHP species, whereas *qnrS1* and *aac(3″)-IId* genes were identified in two of out five NHP species.

## 3. Discussion

Antimicrobial resistance in foodborne pathogens represents a critical global health challenge, with wild animals serving as key contributors in their transmission. A report by Antimicrobial Resistance Collaborators indicated that AMR is projected to directly cause over 39 million deaths and 169 million related deaths between 2025 and 2050 [58]. This review identified and synthesised information from 37 published studies on AMR phenotypic and genotypic patterns in NHPs.

### 3.1. Prevalence of Antimicrobial Resistance Bacteria in NHPs

Evidence from existing research clearly highlights that NHPs may serve as natural reservoirs and sentinel species for AMR, and are highly likely to act as sources of contamination, thus playing an indispensable role in the transmission of AMR to humans and the environment [59]. This was evident in this review, where we observed several zoonotic pathogens, such as *Staphylococcus* spp., *Escherichia* spp., *Shigella*, *Yersinia,* and *Campylobacter*, were frequently acquired from NHPs. Among them *Staphylococcus* spp., and *Escherichia* spp., particularly *E. coli*, were common among all five species of NHPs. *Staphylococcus* species are important antibiotic-resistant bacteria, and *Staphylococcus aureus*, especially MRSA (methicillin-resistant *S. aureus)*, is a serious public health concern on a worldwide scale [60]. *S. aureus* carriers are at higher risk of infection and they are presumed to be an important source of the spread of S. aureus strains among individuals [61]. *Escherichia* spp., particularly *E. coli*, are significant causes of antibiotic-resistant infections, posing a major public health concern due to their ability to acquire and spread resistance genes, leading to multidrug-resistant (MDR) strains [62]. *E. coli* is the most common pathogen leading to uncomplicated cystitis and also results in other extraintestinal illnesses, including pneumonia, bacteremia, and abdominal infections such as spontaneous bacterial peritonitis [63]. Wild animals, including non-human primates (NHPs), are generally not exposed to antibiotics; however, they can acquire antibiotic-resistant bacteria, such as ESBL-producing *E. coli*, through foraging and drinking in natural environments contaminated by anthropogenic sources. Studies have reported that NHPs serve as hosts for the pathogens MRSA and ESBL-producing *Escherichia coli* [64,65]. Bacteria that colonise NHPs can spread clinically relevant ARBs and ARGs across the “human-animal-ecosystem” health interface once they have acquired AMR. In the natural–private–urban interface, NHPs increasingly come into contact with humans and livestock, have a primarily omnivorous diet, inhabit pristine habitats and are consumed as bushmeat, which may facilitate the spread of AMR across different ecological niches. To date, ARBs have been isolated from various NHP species [66,67]. Compared to wild NHPs, captive NHPs have higher AMR prevalence, which is associated with human activities. This is because it may be transmitted through bites, arthropod vectors, aerosols, faecal contamination, and/or the captive keeping of infected NHPs as endangered animals, thereby widely spreading ARB to humans (and vice versa) and the broader environment [68]. Therefore, identifying the potential risks and negative impacts of AMR on NHP populations should remain a priority for disease control.

### 3.2. Prevalence of Antibiotic Resistance in NHPs

Antimicrobial resistance is when a microorganism develops greater or complete resistance to antimicrobials that were once able to treat it. This is mainly driven by the overuse and misuse of antimicrobials in both human and animal settings [69]. This review reported antibiotic resistance of bacteria isolates for NHPs. Among the tested antibiotics, Tetracycline, Ciprofloxacin, and Erythromycin showed resistance in almost all the NHP species. Tetracycline, ciprofloxacin, and erythromycin are well known antibiotics that are used as broad-spectrum antimicrobial control and thus resistance against them raises an alarm to encourage development of strategies to prevent ARB [70]. In addition, bacteria resistance to tetracycline, ciprofloxacin, and erythromycin indicates multidrug resistance, meaning that NHPs may contain bacteria that are resistant to many different classes of antibiotics, which can make infections more difficult to treat, thus it is important for anti-AMR stakeholders to shift their attention to these NHPs as potential threats to global health.

### 3.3. Prevalence of Antibiotic Resistance Genes in NHPs

The presence of ARGs is the root cause of bacterial resistance. Pathogenic bacteria acquire ARGs through plasmid exchange at the gene level and develop strong resistance to antibiotics [71]. ARGs can spread effectively between cells, including from commensal and non-pathogenic bacterial species to pathogens, thanks to their location on mobile genetic elements (MGEs) like transposons and conjugative elements (like plasmids) [72]. From the review we identified ARGs with public health significance in bacterial isolates from all the NHPs across different geographical regions. Among these ARGs, *blaZ* (β-lactams), *qnrS1* and *gyrA* (fluoroquinolones), *sul2* (sulfonamides) and *aac(3″)-IId* (aminoglycosides) were high in among the NHPs but tet family genes (tetracyclines) were widely distributed geographically. In addition, we observed that the *sul2* (sulfonamides) antibiotic resistant gene was present in bacterial isolates from four out of the five NHP species. These findings indicate that *blaZ* (β-lactams), *qnrS1* and *gyrA* (fluoroquinolones), *sul2* (sulfonamides), tet family genes (tetracyclines), and *aac(3″)-IId* (aminoglycosides) have successfully been established in different NHP species and thus drugs that can target these genes should be developed to reduce their risk.

The AMR of NHPs has negative impacts on animal health, human health, and the environment. Highly fragmented habitats have led to omnivorous NHPs living on the fringes shared by free-range livestock and humans. Wild NHPs acquire drug-resistant bacterial strains through exposure to human food waste, contact with other species, soil, faeces, and/or consumption of contaminated water. For captive and/or endangered rescued NHPs, AMR reduces the efficacy of antibiotics, making the treatment of bacterial infections more challenging. Another negative impact is potential zoonotic ARB transmission from NHPs, posing an occupational hazard exposure and public health threats. A cross-sectional survey by National Resource Center for Non-Human Primates in 2023 revealed that 67.40% of veterinarians had experienced occupational hazard exposure, including biochemical injuries (infectious diseases, allergens, chemical disinfectants, and volatile anaesthetics, etc.) (accounting for 62%), harmful exposures (accounting for 61%), inhalation exposure (accounting for 49%), contact exposure (accounting for 44%), and instrument-related injuries (accounting for 25%), highlighting the significant risks of occupational hazard exposure. Additionally, the ability of *Burkholderia pseudomallei*, *Mycobacterium tuberculosis*, and *Yersinia* to spread among healthcare workers poses a public health threat [73]. According to a study conducted by the New Mexico Department of Health, the strains of *Shigella flexneri* found in homeless people were highly homologous to the ones in zoo primates and carried mutant genes (parCS80I, gyrAD87N, and gyrAS80I) that are linked to fluoroquinolone resistance. In the same study, bidirectional transmission of multidrug-resistant Shigella flexneri between humans and NHPs was further demonstrated [74]. For interventions, the New Mexico Department of Health proposed that cross-species collaborative prevention and control under the framework of One Health is crucial. They suggested that the strengthening of environmental disinfection in zoos and improving of sanitation facilities for the homeless can help reduce the risk of transmission. In addition, the department banned the use of fluoroquinolones for clinical treatment of multidrug resistant strains and suggested the use of alternative options such as macrolides [74]. Moreover, in Brazil, they have adopted a method of early monitoring of the symbiotic microbiota of these primates to help in early detection of AMR threats [33]. Finally, human–wild-animal contact can increase the risk of zoonotic and vector-borne disease outbreaks by exposing primates to antibiotic-resistant bacteria (ARB) or antibiotic-resistant genes (ARGs) through a variety of pathways. By bridging the gap between contaminated human environments and wildlife, anthropogenic pressures such as urban encroachment, tourism, and deforestation greatly increase the exposure of non-human primates (NHPs) to antibiotics. Ecotourism is a growing sector in many countries due to increasing appreciation and desire to observe and interact with animals in their natural setting, as it provides for more up-close and personal contact between tourists and wild animals [75]. However, there are possible health risks, including the spread of infectious diseases associated with tourists’ ignorance about pathogen transmission and uncontrolled interactions with wildlife (caused by ecotourism operators’ poor management) [76]. Tourists and wild animals may be more likely to spread antibiotic-resistant strains due to their close contact, which may result in the transfer of non-pathogenic bacteria between humans and animals, which in turn impacts the host’s resistance to exogenous bacterial colonisation [77,78]. Deforestation increases human–wild-animal contact, which raises the possibility of zoonotic and vector-borne disease outbreaks [79]. Deforestation creates new standing water sources in the surrounding area (such as flooded areas or water containers), which are ideal breeding grounds for mosquitoes and other disease-carrying insects [80,81]. There is a significant risk of developing new animal reservoirs for disease and innovative pathways for transmission due to the opportunistic mosquito’s rapid population growth and ability to adapt to feed on new animals. All these factors reveal the serious impact ARGs and ARBs in wildlife have on humans, thus we suggest that all stakeholders involved should consider integration of wildlife AMR monitoring into national and global AMR action plans.

## 4. Limitations

In summary, this is the first systematic review to integrate phenotypic and genotypic patterns of antimicrobial resistance in NHPs and identify knowledge gaps. We conducted a comprehensive literature search using well-defined selection criteria and systematically synthesised the data. Unfortunately, this systematic review has potential limitations. For example, it is limited to articles published in English, which may result in the exclusion of relevant studies in other languages. Another limitation is that most sampling studies were conducted between 2001 and 2019. Traditional culture-based antimicrobial susceptibility testing methods were primarily used, which are not the preferred methods for reporting clinical bacterial testing and AMR surveillance in current practice. Finally, the presence of heterogeneity (including differences in bacterial targets, sample sources (e.g., clinical vs. environmental), and analytical standards) in this review reduced the feasibility of performing more comprehensive quantitative syntheses.

## 5. Methodology

### 5.1. Aim and Research Questions

This study undertook an extensive examination of the literature on AMR in NHPs by reviewing the various studies that investigated the prevalence of AMR in NHPs between 2015 and 2025 inclusively. The following research questions outline the specific areas that were explored in the articles:

(A) What is the prevalence of AMR in NHPs?
(B) What is the current status of research into phenotypic and genotypic patterns of antimicrobial resistance in NHPs?
(C) What are the technologies being used in detecting AMR in NHPs?

### 5.2. Data Sources and Search Strategy

We conducted a systematic review in accordance with the Preferred Reporting Items for Systematic Reviews and Meta-Analyses (PRISMA) guidelines [82]. The following databases were searched: PubMed (National Library of Medicine-NLM), Scopus (Elsevier), Web of Science Core Collection (Clarivate Analytics), Springer Link (Springer), and Science Direct (Elsevier) bibliographic databases. We used the following string to search for relevant full-text articles individually, i.e., (“Non-human primates” OR Primate*) AND ((Antibiotic resistance) OR (Antimicrobial resistance) OR (Resistance genes) OR (Bacterial)), Detailed information on each database search strategy is available at Supplementary File S1. All studies were published online in English between 1 January 2015 and 1 January 2025. 

### 5.3. Eligibility Criteria

This review aimed to identify peer-reviewed studies on the presence and/or potential transmission of AMR in non-human primates (NHPs) from the *Cebinae*, *Callithrix*, *Hominidae*, *Cercopithecidae,* and *Atelidae* species. Therefore, studies meeting at least one of the following criteria were included in this systematic review: (i) studies published online between 1 January 2015 and 1 January 2025; (ii) studies that isolated bacteria from NHPs and assessed phenotypic resistance to specific antibiotics and/or (iii) studies that identified ARGs in bacteria isolated from NHPs; (iv) studies exclusively focused on NHPs and drug-resistant bacteria; (v) articles written in English. Exclusion criteria: (i) articles focusing on topics other than AMR in NHPs; (ii) studies containing duplicate data or overlapping articles; (iii) conference abstracts, review articles/meta-analyses, and articles published before January 2015, as we consider 2015 to be the starting point for data collection, since most relevant records began in 2015; (iv) non-English articles [83].

### 5.4. Selection of Studies and Data Extraction

The extracted data were independently conducted by two authors (J.W. and S.K.O.) and verified by other authors. Disagreements were resolved through discussion, and data were extracted from the selected articles and used to create a comprehensive database which was summarised and organised into columns by the following themes, including (a) species; (b) locations; (c) continent; (d) life context; (e) year; (f) type of sample; (g) resistant bacteria; (h) antibiotic resistance; (i) detection test; (j) resistance genes; and (k) reference. Detailed summaries are provided in Table 1, Table 2, Table 3, Table 4 and Table 5.

## 6. Conclusions and Future Directions

To the best of our knowledge, AMR has become a global threat that must be addressed in both human and veterinary medicine. NHPs carrying zoonotic pathogens have garnered significant attention from scholars due to their importance as hosts and vectors, particularly in relation to human health. In this review we conclude that *Staphylococcus* spp. and *Escherichia* spp. are the most common pathogen bacteria spp. among NHPs, whereas *blaZ* (β-lactams), *qnrS1* and *gyrA* (fluoroquinolones), *sul2* (sulfonamides), tet family genes (tetracyclines) and *aac(3″)-IId* (aminoglycosides) are the most prevalent antimicrobial genes identified in pathogenic bacteria isolated from these NHPs, thus there is a need to give more research attention to these bacteria spp. and resistance genes to develop effective strategies that will prevent the spread of these ARBs/ARGs to humans and animals. Furthermore, these results therefore confirm NHPs as potential natural reservoirs of AMR, making NHPs an indicator species for monitoring the spread of ARB. Additionally, the role of the environment in the emergence and spread of drug-resistant bacteria should not be underestimated. Rapid and sensitive determination of drug resistance in both cultivable and uncultivable bacteria should be achieved through the combination of multilocus sequence typing, whole-genome sequencing, whole-metagenome sequencing, and machine learning algorithms. In addition, we suggest that governments should establish monitoring systems for the prevalence of antibiotic resistance in bacteria sourced from NHPs to effectively control the spread of ARB and thereby improve animal welfare.

## Figures and Tables

**Table 1:** Antibiotic resistance in *Cebidae*, including species, locations, life context, year, type of sample, resistant bacteria, antibiotic resistance, and resistance genes.

Species	Locations	Life Context	Type of Sample	Resistant Bacteria	Antibiotic Resistance	Detection Test	Resistance Genes	Ref
Black-horned capuchin monkey (*Sapajus nigritus*)	Brazil	Wild	Oral and rectal swabs	Enterobacteriaceae	AMX, AMP, FOX, IPM	ND	ND	[21]
	Brazil	Captive and wild	Faeces	*E. durans, E. faecium, E. faecalis*, *E. hirae*	RFP, TC, E, CI, NFX, CAP, AMP	PCR	*msrC*, *tetM, tetL*	[22]
	Brazil	Captive	Oral, ocular, nasal swabs	*Streptococcus* spp. *Staphylococcus* spp.	GM, ENR, CTX, PG, OX	PCR	*mecA*	[23]
Central American squirrel monkey (*Saimiri oerstedii*)	Costa Rica	Captive	Faeces	*Salmonella enterica*	CI, NIT	ND	ND	[24]

**Note**—AMX: Amoxicillin; AMP: Ampicillin; FOX: Cefoxitin; IPM: Imipenem; RFP: Rifampicin; TC: Tetracycline; E: Erythromycin; CI: Ciprofloxacin; NFX: Norfloxacin; CAP: Chloramphenicol; GM: Gentamicin; ENR: Enrofloxacin; CTX: Cefotaxime; PG: Penicillin; OX: Oxacillin; NIT: Nitrofurantoin; ND: Not declared.

**Table 2:** Antibiotic resistance in *Hominidae*, including species, locations, life context, year, type of sample, resistant bacteria, antibiotic resistance, and resistance genes.

Species	Locations	Life Context	Type of Sample	Resistant Bacteria	Antibiotic Resistance	Detection Test	Resistance Genes	Ref
Sumatran orangutan (*Pongo abelii*)	Indonesia	Wild	Faeces	*K. pneumoniae*	AMP, NA, SM	PCR	*bla_TEM_, bla_SHV_, bla_CTX-M_, tetA*	[25]
Chimpanzee (*Pan troglodytes*)	Senegal	Wild	Faeces	*E. coli*, *Enterobacter* spp.	CTX, COL, ETP	WGS	*oqxA, oqxB, fosA, bla_CMH-3_, bla_ACT-6_, bla_CMG1,_* *bla_CTM-X_* *_,_* *qnrS1, sul2, tetA*	[26]
Mountain gorilla (*Gorilla beringei beringei*)	Uganda	Wild	Faeces	*E. coli*	TMP-SMX, CI, GM, EFT	PCR	*dfrA, aadA, bla_OXA_, catB*	[27]

**Note**—Ampicillin: AMP; Nalidixic acid: NA; SM: Streptomycin; CTX: Cefotaxime; COL: Colistin; ETP: Ertapenem; TMP-SMX: Trimethoprim-Sulfamethoxazole; CI: Ciprofloxacin; GM: Gentamicin; EFT: Ceftiofur; WGS: Whole genome sequencing.

**Table 3:** Antibiotic resistance in *Callithricidae*, including species, locations, life context, year, type of sample, resistant bacteria, antibiotic resistance, and resistance genes.

Species	Locations	Life Context	Type of Sample	Resistant Bacteria	Antibiotic Resistance	Detection Test	Resistance Genes	Ref
Common marmoset (*Callithrix jacchus*)	United States	Captive	Oral, skin, swab, faeces	*S. aureus, Yersinia* spp. *Campylobacter* spp.	PRM, NIT, OX	ND	ND	[28]
Mustached tamarin (*Saguinus mystax*)	Peru	Semi-captive	Faeces	*E. coli*	CAP, GM	WGS	*bla_CTM-X_, floR, catA, cmlA1, * *aac(3″)-IId, * *aadA, aph(6″)-Id, aph(3″)-IIa, qnrB* *, qnrS1* *, sul1, sul2, sul3, tetA, tetB, tetM*	[29]
Red-handed tamarin (*Saguinus midas*)	Brazil	Captive	Exudate	*Klebsiella pneumoniae*	AMX, PG, NV, FLR, SUD, SMX	ND	ND	[30]
Black-tufted marmoset (*Callithrix pencillata*)	Brazil	Wild	Faeces	*Staphylococcus epidermidis*	PG, FOX, CI, CLI, E	ND	ND	[31]
Silvery marmoset (*Mico argentatus*)	England	Captive	Tissues	*Yersinia pseudotuberculosis*	CLI	ND	ND	[32]
Golden lion tamarin (*Leontopithecus rosalia*)	Brazil	Wild	Oral and rectal swabs	*Staphylococcus* spp.	PG, E, OX, FD	ND	ND	[33]

Note—PRM: Paromomycin; NIT: Nitrofurantoin; OX: Oxacillin; CAP: Chloramphenicol; GM: Gentamicin; AMX: Amoxicillin; PG: Penicillin; NV: Novobiocin; FLR: Florfenicol; SUD: Sulfadimethoxine; SMX: Sulfathiazole; FOX: Cefoxitin; CI: Ciprofloxacin; CLI: Clindamycin; E: Erythromycin; FD: Fusidic acid; ND: Not declared.

**Table 4:** Antibiotic resistance in *Cercopithecidae*, including species, locations, life context, year, type of sample, resistant bacteria, antibiotic resistance, and resistance genes.

Species	Locations	Life Context	Type of Sample	Resistant Bacteria	Antibiotic Resistance	Detection Test	Resistance Genes	Ref
Long-tailed macaque (*Macaca fascicularis*)	Belgium	Captive	Gastric mucosa	*Helicobacter suis*	ENR, LVX, MFX, SH, MY, TC	WGS	*gyrA, acrB*	[34]
	China, Indonesia	Captive	Faeces	*Campylobacter coli,* *Campylobacter jejuni*	TC, E, CI, AMX	ND	ND	[35]
	United States	Captive	Nasal swab	MRSA, VRSA, VISA	FOX, TMP-SMX, CLI, E, PG, VAN	ND	ND	[36]
	United States	Captive	Nasal swab	MSSA, MRSA	ND	WGS	*blaZ, tet38, aph*(*3*″)*-IIa, gyrA*	[37]
	Brunei Darussalam	Wild	Faeces	*Staphylococcus* spp.	TMP, SMZ, FD	ND	ND	[38]
Assamese macaque (*Macaca assamensis*)	Nepal	Wild	Oral	MRSA	PEN, OX, FOX, GM, E	DNA Microarray	*blaZ, aacA-aphD, aph*(*3*″)*-IIa, erm*(*C*)*, mph*(*C*)*, dfrA, msrA*	[39]
Toque macaque (*Macaca sinica*)	Sri Lanka	Wild	Faeces	*Campylobacter* spp. *Salmonella* spp.	TC, CI, NA	ND	ND	[40]
Rhesus macaque (*Macaca mulatta*)	United States	Captive	Cephalic chambers	*E. faecalis*	CI, ENR, TMP-SMX, TC, CAP, B, E	WGS	*bcrA, bcrB, bcrR, catA, catB, gyrA, aph*(*3*′)*-II a, tetM, tetS, tetL, dfrG*	[41]
	Nepal	Wild	Saliva	MRSA	ND	DNA microarray	*aacA-aphD, dfrA, ermC, aph*(*3*″)*-IIa, blaZ, mecA, msrA*	[42]
	United States	Captive	Faeces	*Shigella flexneri*	AMP, AMC, GM, TC, CI, ENR, LVX, NA	WGS	*aadA, aac*(*3*″)*-IId, bla_OXA_, oqxA, oqxB, catA, tetB, bla_TEM_, qnrS1, bla_CTX-M_*	[43]
	Nepal	Wild	Saliva	MRSA	CI, GM, E	WGS	*gyrA, ermC, aacA-aphD, blaZ*	[44]
	China	Captive	Faeces, tissue fluid	*E. coli, K. pneumoniae, P. mirabilis*	FLR, TC, KM, AMP, IPM, FOX, SM	ND	ND	[45]
	United States	Captive	Rectal swab	*Campylobacter jejuni,* *Campylobacter coli*	CI, AZM, CLI, TC	WGS	*aph*(*3*″)*-IIa, gyrA, tetO, floR, sul2*	[46]
	China	Captive	Faeces	*E. coli, P. mirabilis, K. pneumoniae*	LVX, ENR, CTX	WGS	*qnrS1, bla_SHV_, bla_TEM_, bla_CTX-M_, sul2, floR*	[47]
	Bangladesh	Wild	Faeces	*Salmonella* spp. *Staphylococcus*	TC, AZM, TMP-SMX, NA, AMP, MET, CLI, RFP	ND	ND	[48]
Vervet monkeys (*Chlorocebus pygerythrus*)	South Africa	Wild	Faeces	*Escherichia fergusonii*	POL, COL, AMK	ND	ND	[49]
	Uganda	Wild	Nasal swab	MRSA	TC, SMZ-TMP, PG	ND	ND	[50]
Golden snub-nosed monkeys (*Rhinopithecus roxellana*)	China	Captive	Faeces	*E. coli*	DOX, TC	PCR	*tetA*	[51]
	China	Captive	Faeces	*Streptococcus agalactiae*	E, TC, CLI	WGS	*mreA, tetM, tet*(*L*)*, tet*(*O*)	[52]
African green monkeys (*Chlorocebus sabaeus*)	Nigeria	Captive	Faeces	*E. coli*	PIP, LVX, TMP-SMX	PCR	*qnrD, qnrA, qnrB, qnrS1*	[53]
	Saint Kitts and Nevis	Captive and wild	Nasal swab	MSSA, MRSA	ND	WGS	*mecA, blaZ, mphC, dfrG, ermC, tetK*	[54]
Olive Baboons (*Papio anubis*)	Ghana	Wild	Oral and rectal swabs	*E. coli,* *Staphylococcus* spp.	PEN, OX	ND	ND	[55]
Guinea baboons (*Papio papio*)	Gambia	Wild	Faeces	*E. coli*	AMK, TMP-SMX, CI, CTX, TC	WGS	*blaEC, aadA, tetA*	[56]

**Note**—MRSA: Methicillin-resistant *Staphylococcus aureus*; VRSA: Vancomycin-resistant *S. aureus*; VISA: Vancomycin-intermediate *S. aureus*; MSSA: Methicillin-susceptible *S. aureus*; E; NR: En rofloxacin; LVX: Levofloxacin; NFX: Norfloxacin; SH: Spectinomycin; MY: Lincomycin; TC: Tetracycline; E: Erythromycin; CI: Ciprofloxacin; AMX: Amoxicillin; FOX: Cefoxitin; TMP-SMX: Trimethoprim-Sulfamethoxazole; CLI: Clindamycin; PG: Penicillin; VAN: Vanc omycin; TMP: Trimethoprim; SMX: Sulfamethoxazole; FD: Fusidic acid; PEN: Penicillin G; OX: Oxacillin; GM: Gentamicin; E: Erythromycin; N A: Nalidixic acid; C AP: Chloramphenicol; B: Bacit racin; AMP: Amp icillin; AMC: Amoxicillin-Clavulanic; FLR: Florfenicol; KM: Kanamycin; KZ: Cefazolin; OFL: Ofloxacin; IPM: Imipenem; SM: Streptomycin; AZM: Azithromycin; CTX: Cefotaxime; MET: Methicillin; RFP: Rifampicin; POL: Polymyxin B; COL: Colistin; AMK: Amikacin; SMZ-TMP: Sulfamethoxazole-Trimethoprim; DOX: Doxycycline; PIP: Piperacillin; ND: Not declared.

**Table 5:** Antibiotic resistance in *Atelidae*, including species, locations, life context, year, type of sample, resistant bacteria, antibiotic resistance, and resistance genes.

Species	Locations	Life Context	Type of Sample	Resistant Bacteria	Antibiotic Resistance	Detection Test	Resistance Genes	Ref
Black howler monkeys (*Alouatta pigra*)	Mexico	Wild	Faeces	*E. coli*	ND	PCR	*sul1, sul2, tetB, * *bla_TEM_*	[57]

**Note**—ND: Not declared.

## Data Availability

Not applicable.

## Supplementary Materials

The following supporting information can be downloaded at: https://www.mdpi.com/article/10.3390/antibiotics14100985/s1, File S1: Detailed information on database search strategy.
